# 

**Published:** 1892-12

**Authors:** 


### Society Meetings.

The next meeting of the Buffalo Microscopical Club will be held Monday evening, December 12, 1892, in the lower lecture-room of the Buffalo Library building. The programme includes a microscopical exhibition on Diseases of the Kidney, and an essay on Some Recent Advances in Water Analysis, and the Use of the Microscope for the Detection of Sewage Contamination, by George W. Rafter, of Rochester, N. Y. Physicians are cordially invited to attend.

The next stated meeting of the Buffalo Academy of Medicine will be held at the Academy rooms, 639 Main street, third floor, Tuesday evening, December 6, 1892, at 8.30 o'clock. The section on Surgery has prepared the following programme: Operations upon the Kidney, by Herman Mynter, M. D.; Actinomycosis Hominis, by Roswell Park, M. D. All members of the Academy are earnestly requested to be present.

### BOOKS RECEIVED.

International Clinics: A Quarterly of Clinical Lectures on Medicine, Surgery, Gynecology, Pediatrics, Neurology, Dermatology, Laryngology, Ophthalmology, and Otology. By professors and lecturers in the leading medical colleges of the United States, Great Britain, and Canada. Edited by John M. Keating, M. D., Philadelphia, Consulting Physician for Diseases of Women to St. Agnes' Hospital, Philadelphia; editor "Cyclopedia of the Diseases of Children." J. P. Crozer Griffith, M. D., Philadelphia, Clinical Professor of Diseases of Children in the University of Pennsylvania; Professor of Clinical Medicine in the Philadelphia Polyclinic. J. Mitchell Bruce, M. D., F. R. C. P., London, England, Physician and Lecturer on Therapeutics at the Charing Cross Hospital. David W. Finlay, M. D., F. R. C. P., Aberdeen, Scotland, Professor of the Practice of Medicine in the University of Aberdeen; Physician to and Lecturer on Clinical Medicine in the Aberdeen Royal Infirmary; Consulting Physician to the Royal Hospital for Diseases of the Chest, London. January, 1892. Philadelphia: J. B. Lippincott Company. 1892.

Anatomy. A Manual for Students and Practitioners. By Fred J. Brockway, M. D., Assistant Demonstrator of Anatomy, College of Physicians and Surgeons, New York, and A. O'Malley, M. D., Instructor in Surgery, New York Polyclinic. Series edited by Bern B. Gallaudet, M. D., Demonstrator of Anatomy, College of Physicians and Surgeons, New York; Visiting Surgeon, Bellevue Hospital, New York. Philadelphia: Lea Brothers & Co.

Accidents and Emergencies. A Manual of the Treatment of Surgical and Medical Emergencies in the Absence of a Physician. By Charles W. Dulles, M. D., Fellow of the College of Physicians, Philadelphia, and of the Academy of Surgery; Physician of the Rush Hospital; formerly Surgeon to the Out-door Department of the Hospital of the University of Pennsylvania, and of the Presbyterian Hospital, in Philadelphia, and Assistant Surgeon, Second Regiment, N. G., Pa. Fourth edition, thoroughly revised and enlarged, with new illustrations. Philadelphia: P. Blakiston, Son & Co., No. 1012 Walnut Street. 1892.

A Pocket Medical Dictionary, giving the Pronunciation and Definition of about 12,000 of the Principal Words used in Medicine and the Collateral Sciences. By George M. Gould, A. M., M. D., Author of a

New Medical Dictionary; Ophthalmic Surgeon to the Philadelphia Hospital. Including a very complete table of the Arteries, Muscles, Nerves, Bacteria, Bacilli, Micrococci, Spirilli, and Thermometric Scales, and dose list of drugs and their preparations, in both the English and metric systems of weights and measures. Philadelphia: P. Blakiston, Son & Co. 1892.

Diseases of the Kidneys and Bladder. A Text-book for Students of Medicine. By W. F. McNutt, M. D., M. R. C. S., Ed., L. R. C. P., Ed., Professor of the Principles of Practice of Medicine, University of California; Professor of the Diseases of the Kidneys and Heart, Post-Graduate Department of the University of California; Consulting Physician and Surgeon to St. Mary's Hospital, and to the Children's Hospital; Member of the International Medical Congress, of the American Medical Association, and of the California State Medical Society; President of the California Gynecological Society; Ex-Acting Assistant Surgeon United States Navy, etc. Philadelphia: J. B. Lippincott Co. 1893.

Manual of Practical Medical and Physiological Chemistry. By Charles E. Pellew, E. M., Demonstrator of Physics and Chemistry in the College of Physicians and Surgeons, (Medical Department of Columbia College,) New York. Honorary Assistant in Chemistry at the School of Mines, Columbia College, etc. With illustrations. New York: D. Appleton & Co. 1892.

A Manual of the Practice of Medicine, prepared especially for Students. By A. A. Stevens, A. M., M. D., Instructor of Physical Diagnosis in the University of Pennsylvania, and Demonstrator of Pathology in the Woman's Medical College, Philadelphia. Illustrated. Philadelphia: W. B. Saunders, 913 Walnut Street, Philadelphia. 1893.

Fissure of the Anus and Fistula in Ano. By Lewis H. Adler, Jr., M. D., Instructor in Diseases of the Rectum in the Philadelphia Polyclinic and College for Graduates in Medicine. Physicians' Leisure Library. Price, 25 cents. George S. Davis. Detroit, Mich. 1892.

International Pocket Visiting List. Sixty patients each week. 1893. Arranged for the use of practitioners. By J. C. Wilson, M. D., Physician to the German Hospital, etc., etc. Philadelphia: J. B. Lippincott Company.

Histology, Pathology, and Bacteriology. A Manual for Students and Practitioners. By Benett S. Beach, M. D., Lecturer on Histology, Pathology, and Bacteriology, New York Polyclinic. Series edited by Bern B. Gallaudet, M. D., Demonstrator of Anatomy, College of Physicians and Surgeons, New York; Visiting Surgeon, Bellevue Hospital, New York. Philadelphia: Lea Brothers & Co.

Transactions of the Washington Obstetrical and Gynecological Society. Volume III. June 7, 1889, to May 16, 1890. Founded October 7, 1882.

---

Notice to Contributors.—We are glad to receive contributions from every one who knows anything of interest to the profession. Articles designed for publication in the Journal should be handed in before the first day of the month. The Editors are not responsible for the views or opinions of contributors. All communications should be addressed to the Managing Editor, 284 Franklin St., Buffalo, N. Y.

